# Lack of Parkinsonian Pathology and Neurodegeneration in Mice After Long-Term Injections of a Proteasome Inhibitor in Olfactory Bulb and Amygdala

**DOI:** 10.3389/fnagi.2021.698979

**Published:** 2021-10-21

**Authors:** Natalia Lopez-Gonzalez del Rey, Tiziano Balzano, Lucia Martin-Rodriguez, Constanza Salinas-Rebolledo, Ines Trigo-Damas, Alejandro Rojas-Fernandez, Lydia Alvarez-Erviti, Javier Blesa

**Affiliations:** ^1^HM CINAC (Centro Integral de Neurociencias Abarca Campal), Hospital Universitario HM Puerta del Sur, HM Hospitales, Madrid, Spain; ^2^Centro de Investigación Biomédica en Red Sobre Enfermedades Neurodegenerativas (CIBERNED), Madrid, Spain; ^3^PhD Program in Neuroscience, Autonoma de Madrid University-Cajal Institute, Madrid, Spain; ^4^Faculty of Medicine, Institute of Medicine, Universidad Austral de Chile, Valdivia, Chile; ^5^Laboratory of Molecular Neurobiology, Center for Biomedical Research of La Rioja (CIBIR), Logroño, Spain

**Keywords:** Parkinson’s disease, proteasome inhibition, lactacystin, neuroinflammation, neurodegeneration

## Abstract

Proteinaceous inclusions, called Lewy bodies (LBs), are used as a pathological hallmark for Parkinson’s disease (PD). Recent studies suggested a prion-like spreading mechanism for α-synucleinopathy where early neuropathological deposits occur, among others, in the olfactory bulb (OB) and amygdala. LBs contain insoluble α-synuclein and many other ubiquitinated proteins, suggesting a role of protein degradation system failure in PD pathogenesis. Therefore, we wanted to study the effects of a proteasomal inhibitor, lactacystin, on the aggregability and transmissibility of α-synuclein in the OB and amygdala. We performed injections of lactacystin in the OB and amygdala of wild-type mice. Motor behavior, markers of neuroinflammation, α-synuclein, and dopaminergic integrity were assessed by immunohistochemistry. Overall, there were no differences in the number of neurons and α-synuclein expression in these regions following injection of lactacystin into either the OB or amygdala. Microglial and astroglial labeling appeared to be correlated with surgery-induced inflammation or local effects of lactacystin. Consistent with the behavior and pathological findings, there was no loss of dopaminergic cell bodies in the substantia nigra and terminals in the striatum. Our data showed that long-term lactacystin injections in extra nigrostriatal regions may not mimic spreading aspects of PD and reinforce the special vulnerability of dopaminergic neurons of the substantia nigra pars compacta (SNc).

## Introduction

Although it is the second most prevalent neurodegenerative disorder worldwide, the primary cause of Parkinson’s disease (PD) is currently unknown (Del Rey et al., 2018). The appearance of classical PD motor symptoms, such as tremor, rigidity, and bradykinesia, are intrinsically associated with loss of dopaminergic neurons in the substantia nigra pars compacta (SNc) and subsequent depletion of dopamine (DA) in the striatum. Other non-motor features, such as rapid eye movement sleep behavior disorder, constipation, and hyposmia, are also frequent, although the correlation between these clinical manifestations and pathological findings is far from straightforward.

Parkinson’s disease is also neuropathologically characterized by the presence of insoluble proteinaceous inclusions named Lewy bodies (LBs) and Lewy neurites, consisting of a wide range of proteins, such as α-synuclein (α-syn), ubiquitin, and neurofilaments (Spillantini et al., 1998). Lewy pathology is found in many brain regions and has been suggested to follow a progressive and predictable staging pattern (Braak et al., 2003) where Lewy pathology would appear first in regions such as the dorsal motor nucleus of the vagus, olfactory bulb (OB) and amygdala. This would fit well with the manifestation of early non-motor symptoms such as constipation or olfactory dysfunction. Then, α-syn would spread in a prion-like manner until it reached the SNc. This prion-like hypothesis is based on different conformational changes through a seeding mechanism and subsequent spreading process (Goedert, 2015). Considerable *in vitro* and *in vivo* data regarding α-syn spreading have been obtained in several species, and several mechanisms have been proposed for axonal and transneuronal transport ultimately leading to nigrostriatal degeneration (Luk et al., 2012a,b; Mougenot et al., 2012; Masuda-Suzukake et al., 2013; Recasens et al., 2014; Sacino et al., 2014; Paumier et al., 2015; Peelaerts et al., 2015; Dehay et al., 2016; Trigo-Damas et al., 2018; Kim et al., 2019). Usually, α-syn pathology appears in brain regions anatomically connected to the injection site. Noteworthy, several studies in rodents have demonstrated neural transmission from or to the OB and amygdala after exogenous and intracerebral injection of a-syn from different species (Mason et al., 2016; Rey et al., 2016, 2018, 2019; Cersosimo, 2018; Niu et al., 2018; Burtscher et al., 2020; Kulkarni et al., 2020; Stoyka et al., 2020).

However, the basis of α-syn aggregation is undetermined. Several neurodegenerative diseases, such as PD, are characterized by proteasomal dysfunction, which results in the accumulation of aggregation-prone proteins. Thus, proteasomal dysfunction, which leads to aberrant protein turnover and buildup of misfolded or damaged proteins, might be linked to the accumulation of α-syn and other proteins in patients with PD (McNaught and Jenner, 2001; McNaught et al., 2001; Martins-Branco et al., 2012; Poewe et al., 2017). Consistent with this hypothesis, proteasome inhibition *in vivo* and *in vitro* reproduces key hallmarks of PD neuropathology namely, LB-like inclusions and neurodegeneration (Bedford et al., 2008; Ebrahimi-Fakhari et al., 2011; McKinnon et al., 2020). Particularly, lactacystin, a proteasomal inhibitor, causes accumulation of α-syn and death of cells *in vitro* (Machiya et al., 2010; Bir et al., 2014; Bentea et al., 2017). Injection of lactacystin to the SNc, striatum or medial forebrain bundle (MFB) induces a PD-like motor phenotype in mice, rats, and pigs that is correlated with loss of dopaminergic neurons in the SNc and dopaminergic terminals in the striatum, striatal DA depletion, and α-syn upregulation in neurons (McNaught et al., 2002a; Ahn and Jeon, 2006; Niu et al., 2009; Vernon et al., 2010; Lorenc-Koci et al., 2011; Mackey et al., 2013; Bentea et al., 2015, 2017; Pienaar et al., 2015a,b; Elson et al., 2016; Savolainen et al., 2017; Lillethorup et al., 2018a). However, despite accumulating evidence for the possible role of the proteasome system in α-syn aggregation, relatively few studies have examined its role in extra nigrostriatal regions such as the OB or amygdala.

Considering all these facts, we hypothesized whether injection of lactacystin would lead to similar synucleinopathy in the OB and amygdala as described in the SNc. The concrete objectives of this study were to analyze behavioral changes, α-syn, and neuronal and glial (microglia and astroglia) involvement after lactacystin injections in the OB and basolateral amygdala (BLA) of wild-type (WT) mice.

## Materials and Methods

### Experimental Design

Thirty-six male C57BL6/C mice (9 weeks old at the start of the experiments) were purchased from Charles River. All procedures involving animals were carried out in accordance with the European Communities Council Directive (2010/63/UE) and Spanish legislation (RD53/2013) on animal experiments and had approval from the Ethical Committee on Animal Welfare and Care for our institution. All efforts were made to minimize suffering and pain in the animals; thus, all the animals were monitored throughout the whole study. The animals were randomly distributed to cages by a technician of the animal facilities. Before any procedure, the cages were randomized to each group by a person not involved in the study. The animals were randomly divided into six groups (six mice per group), with three of them assigned as AMY: AMY SHAM (mice injected in the left BLA with 0.9% NaCl), AMY LAC-3M (mice injected in the left BLA with lactacystin (LAC) and sacrificed at 3 months), and AMY LAC-6M (mice injected in the left BLA with LAC and sacrificed at 6 months) groups. The remaining three were assigned as the OB: OB SHAM (mice injected in the left OB with 0.9% NaCl), OB LAC-3M (mice injected in the left OB with LAC and sacrificed at 3 months), and OB LAC-6M (mice injected into the left OB with LAC and sacrificed at 6 months) groups. All the experiments were blinded, and investigators responsible for data collection and analysis were blinded. The animals were left to acclimate for 15 days before basal behavioral assessments, and 15 days after the surgery, postoperative behavioral analysis was conducted. The animals were sacrificed at various time points 3 or 6 months post injection. The sham animals were sacrificed at 6 months of age.

### Unilateral Lactacystin Injection in the Olfactory Bulb or Basolateral Amygdala

The surgery was carried out on male C57/BL6/J mice weighing 27–35 g at the start of the experiment. The mice were anesthetized with a solution of ketamine 90 mg/kg and xylazine 9 mg/kg diluted in 0.9% NaCl. The solution was injected at a concentration of 0.02 ml/g.

The animals were secured in a stereotaxic frame and injected with lactacystin in the left hemisphere (Cayman, reference 70980) at a final concentration of 2 μg/μl (in 0.9% NaCl). The lactacystin solution was always prepared fresh before the surgery and maintained on ice. The coordinates of injection were as follows: (mm from bregma and dura) for the OB (2 μl with a rate of 0.5 μl/ml; coordinates AP + 4,5; ML + 0,75; DV -1) and for the BLA (1 μl with a rate of 0.2 μl/ml; AP -1,4; ML + 2,6; DV -4,5) (Franklin and Paxinos, 2003). The sham animals were only injected with 0.9% NaCl. After each injection, the needle was left in place for 5 min and then slowly withdrawn. To evaluate the long-term effect of lactacystin injection, immunohistochemistry against ubiquitin was performed and quantified as detailed in sections “Brain Immunohistochemistry” and “Immunohistochemical Quantification in the Basolateral Amygdala and Olfactory Bulb.” No increased levels of ubiquitin in the BLA or OB were observed after 3 and 6 months (Supplementary Figure 1).

### Fixation and Tissue Processing

The mice were deeply anesthetized with a lethal dose of sodium pentobarbital and transcardially perfused with 0.9% saline followed by 4% paraformaldehyde in 0.1 M phosphate buffer (pH 7.4). Whole brains were removed and post-fixed in the same fixative solution overnight at 4°C. The brains were cryoprotected in a 30% sucrose solution. The solution was changed every 3 days until the brains sank and were ready for processing. This process lasted around 1 week; if the brains were not immediately processed, they were stored in a cryoprotected solution at −20°C. Finally, the brains were cut in the coronal plane on a freezing microtome at 30 μm to produce 10 matched series.

### Brain Immunohistochemistry

Coronal free-floating 30-μm thick sections were washed with Tris buffer (TB) and treated with citrate buffer (pH 6) for 30 min at 37°C for antigen retrieval. The inhibition of endogenous peroxidase activity was performed using a mixture of 10% methanol and 3% concentrated H_2_O_2_ for 20 min. Normal serum from the same species as the secondary antibody (normal goat serum or normal horse serum) was applied for 3 h to block non-specific binding sites.

The sections were immunostained at 4°C for a duration of 72 h using primary antibodies directed against ionized calcium-binding adapter molecule 1 (IBA1, 1:1,000, rabbit; Wako, Osaka, Japan) as microglial marker, glial fibrillary acidic protein (GFAP, 1:500, mouse; Sigma, St. Louis, United States) as an astroglial marker, tyrosine hydroxylase (TH, 1:1,000, mouse; Chemicon, Burlington, United States), α-synuclein (α-syn, 1:1,000, rabbit; Abcam, Cambridge, United Kingdom), phosphoSer129-α-synuclein (pS129-α-syn, 1:4,000, rabbit; Abcam, Cambridge, United Kingdom), and ubiquitin (1:250, rabbit; Sigma, St. Louis, United States). The sections were washed with Tris-buffered saline (TBS) and transferred for 2 h to a solution containing the corresponding secondary biotinylated antibody (goat anti-rabbit, 1:400; Chemicon, Burlington, United States; or horse anti-mouse, 1:400; Vector Laboratories, Burlingame, United States). Then, the sections were incubated for 45 min with the avidin-biotin-peroxidase (PK-6100, ABC Vectastain; Vector Laboratories, Burlingame, United States) complex. Immunohistochemical reactions were visualized by incubating the sections with 0.05% 3, 3′-diaminobenzidine (DAB, Sigma, St. Louis, United States) and 0.003% H_2_O_2_. The sections were then dehydrated with graded ethyl alcohol (EtOH) and cleared in two changes of xylene before being mounted in dibutylphthalate polystyrene xylene (DPX) and applying glass coverslips.

Omission of the primary antibody resulted in non-staining (images not shown).

### Immunohistochemical Quantification in the Basolateral Amygdala and Olfactory Bulb

Images were photographically recorded under an optical microscope (DM 2500; Leica, Wetzlar, Germany) and quantified using the image analysis software Image-Pro Plus 6.0.0.26 (Media Cybernetics, Inc., Rockville, MD, United States).

For the analysis of microglial activation, eight to sixteen 40 × fields (0.074 mm^2^) were used per animal, and the perimeter of microglial cells was quantified as in Hovens et al. (2014). Two sections were used per animal. Briefly, all the images were first converted from pixel into μm using a microscope calibration scale bar. Then, applying an intensity threshold (histogram-based manual intensity range selection) and size filter (area filter range), only the microglial cells were selected. In our case, the threshold of intensity was 0–180, and the size of the filter was 150-infinity μm^2^. The average perimeter of all microglia in the selected area was measured, and the results were expressed in μm, being lower in the amoeboid activated microglia.

The analysis of astrocyte activation was performed on the BLA and OB using the Image-Pro Plus 6.0.0.260 software. Nine randomly selected 10 × fields (1.18 mm^2^) were used per section. Two sections per animal were used. Applying an intensity threshold (0–180) filter, the GFAP + cells were selected, and no filter size was applied. The results were expressed as the percentage of GFAP-covered areas, being higher in the case of astrogliosis.

The analysis of α-syn expression was performed on the BLA and OB using the Image-Pro Plus 6.0.0.260 software. The entire area of the BLA and OB was analyzed by 10 × (covering an area of 1.18 mm^2^) and 5 × (covering an area of 4.72 mm^2^) microscope magnification, respectively. Two sections were used per animal. In both areas, cells expressing α-syn were filtered applying an intensity threshold (0–220) and filter size (10–150 μm^2^). The average optical density for each image was recorded, and the results were normalized to the contralateral control hemisphere.

The analysis of pS129-α-syn and ubiquitin expression was performed on the BLA and OB using the Image-Pro Plus 6.0.0.260 software. The entire area of the BLA and OB was analyzed by 10 × (covering an area of 1.18 mm^2^) and 5 × (covering an area of 4.72 mm^2^) microscope magnification, respectively. Two sections were used per animal. In both areas, the region of interest was outlined using the freehand selection function, the average optical density for each image was recorded, and the results were normalized to the contralateral control hemisphere.

Dopaminergic innervation in the striatum was studied by immunohistochemical localization of TH. The relative optical densities of TH-ir fibers in the striatum were quantified using computer-assisted image analysis techniques (ImageJ 1.41o; National Institutes of Health, Bethesda, United States). Images were captured in black and white 8-bit monochrome using a digital camera (AxioCam HRc; Zeiss, Jena, Germany) attached to a Nikon Multiphot microphotography system (Melville, United States). Digital images were captured under the same exposure settings for all experimental cases. Five rostro-caudal sections, regularly spaced at intervals of 120 μm, were examined for each animal. The optical density of a 0.08-μm^2^ region of white matter in the corpus callosum of the same section was subtracted as background. The percent reduction of each marker was determined as percent loss in treated animals compared with the value of controls in the same region.

### Stereological Analysis of TH Immunopositive Cells in the Substantia Nigra

The total number of dopaminergic (TH+) neurons in the SNc was assessed by stereology with the optical fractionator method in regularly spaced 30-μm thick sections (every forth) covering the entire rostro-caudal axis of the SNc (Izco et al., 2019, 2020). Nine sections were used per animal. This method was carried out using a computer-assisted image analysis system consisting of a microscope (Olympus BX3, Shinjuku, Japan) equipped with a computer controlled motorized stage, a camera, and the StereoInvestigator software (Stereo Investigator 2017; MicroBrightField, Williston, VT, United States). The identification of the SNc region of each section to be counted was outlined at ×2 magnification, and immunolabeled cells were counted with a ×100 oil immersion objective (counting frame, 50 μm × 50 μm; sampling grid, 100–125 μm). The first section was randomly selected. Cells exhibiting a neuronal phenotype, with clear nuclear membrane, distinct nucleolus, and TH immunoreactivity in the cytoplasm were counted through the entire thickness of the tissue by a researcher “blinded” to the treatment regimen of each experimental animal assessed. After the counting was finished, the total number of neurons was automatically calculated by the software using the formula described by West (1993).

### Analysis of Neuronal Density in Nissl-Stained Sections

Sections were processed as previously described and incubated in 70% ethanol overnight. The next day, after a quick wash in distilled water, they were incubated at 45°C in agitation with cresyl violet for 5 min. Following another quick wash in distilled water, the sections were incubated in sequential solutions of 70% ethanol for 1 min, 96% ethanol for 1 min, and chloroform for 10 min (in agitation), and then washed in 100% ethanol. Finally, the slices were incubated in the differentiation solution under visual control and rapidly washed in ethanol 100% before being incubated in clean xylol 9 × 5 min and coverslipped using DPX as a mounting medium.

Neuronal density in the BLA and OB of Nissl-stained sections was quantified using the Image-Pro Plus 6.0.0.260 software. For the BLA, the entire area was photographed by 10 × microscope magnification (covering an area of 1.18 mm^2^). One or two sections were used per animal. Neuronal cells were discriminated from glial cells by first applying an intensity threshold (0–180) and then a filter size (60-infinity μm^2^). For the OB, the entire area was photographed by × 5 microscope magnification (covering an area of 4.72 mm^2^). One or two sections were used per animal. An intensity threshold (0–180) and a filter size (15-infinity μm^2^) were applied to discriminate neuronal cells from glial cells.

In both regions, cell density was quantified using the “count adjusted” function, and the results were expressed in cells/mm^2^.

### Longitudinal Assessment of Motor Coordination and Balance

Motor coordination and balance were assessed using a rotarod apparatus (PanLab/Harvard Apparatus, Barcelona, Spain). First, the mice were trained to walk against the motion of a rotating drum at a constant speed of 12 rotations per minute (RPM) for a maximum of 2 min. In total, the training was performed for 3 days (3 trials per day). Mice falling off during a training trial were put back on the rotating drum. For the test, three trials per day were performed using an accelerating speed level (4–40 RPM) mode of the apparatus for a maximum of 5 min. The apparatus was wiped with a 70% ethanol solution and dried before each trial. The latency to fall off the rotarod was recorded, and the mean of three trials was calculated.

Movement alterations were also assessed by pole test as described in Matsuura et al. (1997). Briefly, the mice were placed with head upward on the top of a vertical rough-surfaced pole (diameter 8 mm; height 57 cm), and the time, until it descended to the floor, was recorded with a maximum duration of 120 s.

For both behavioral tests, a total of 8 tests (1/week) were performed before the surgery (BASAL), and 13 days of tests (1/week) were performed after the surgery (POST). The only two exceptions were on POST1 (the first day of test after the surgery) and POST 9 (the first day of test after the sacrifice at 3 months) when the test was performed 2 weeks after the previous one.

### Statistical Analysis

The results are expressed as the mean ± SEM. All statistical analyses were performed using the GraphPad Prism software. For all immunohistochemistry, a two-way ANOVA and Bonferroni multiple comparisons were performed to determine which pairs were significantly different, and a two-tailed unpaired Student’s *t*-test was performed to determine differences between two hemispheres of the same experimental group. A confidence level of 95% was accepted as significant.

Behavioral data were analyzed by a two-way ANOVA, and Bonferroni multiple comparisons were performed to determine which pairs were significantly different. A confidence level of 95% was accepted as significant.

## Results

### Proteasome Inhibition With Lactacystin in the Basolateral Amygdala or Olfactory Bulb Does Not Impair Balance and Motor Coordination in Mice

**FIGURE 1:**
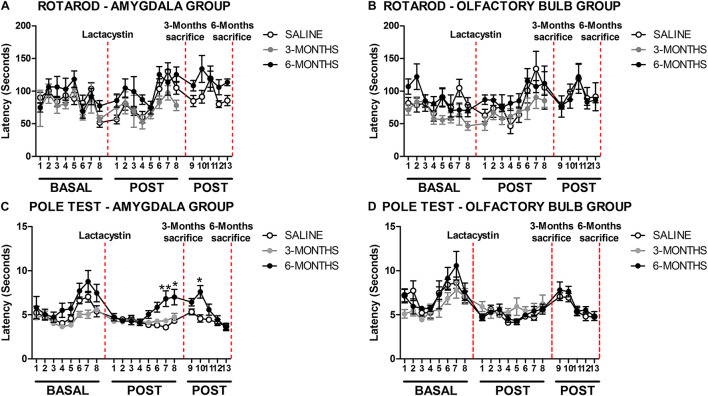
Proteasome inhibition with lactacystin in the basolateral amygdala (BLA) or olfactory bulb (OB) does not impair motor coordination in mice. Motor coordination and balance were assessed by rotarod and pole test in the **(A,C)** BLA and **(B,D)** OB groups. For both behavioral tests, a total of 8 tests (1/week) were performed before the surgery (BASAL), and 13 tests (1/week) were POST. The only two exceptions were on POST1 (the first day of test after the surgery) and POST 9 (the first day of test after the sacrifice at 3 months) when the test was performed 2 weeks after the previous one. Six animals were used per group. A two-way ANOVA and Bonferroni multiple comparisons were performed to determine which pairs were significantly different. A confidence level of 95% was accepted as significant. *p < 0.05 vs. saline group; **p < 0.01 vs. saline group.

The effects of proteasome inhibition with lactacystin (LAC in the figures) in the BLA or OB on balance and motor coordination were assessed by rotarod and pole tests, as detailed in methods. No behavioral changes were observed in the rotarod test between LAC and control groups in both injected regions (Figures 1A,B). A slight but significant difference (*p* < 0.05) was observed in the pole test comparing the LAC-6M and control group mice that were injected in the BLA. The LAC-6M group showed higher latency to climb down in the POST 7, 8, and 10 time points in comparison with the control group (Figure 1C). No differences between the LAC-3M and control groups injected in the BLA were observed in the rotarod test (Figure 1C). Similarly, no differences between the LAC and control groups injected in the OB at any time point were observed in the pole test (Figure 1D).

### Proteasome Inhibition Induces Neuroinflammation but Does Not Alter Neuronal Density in the Basolateral Amygdala and Olfactory Bulb

**FIGURE 2:**
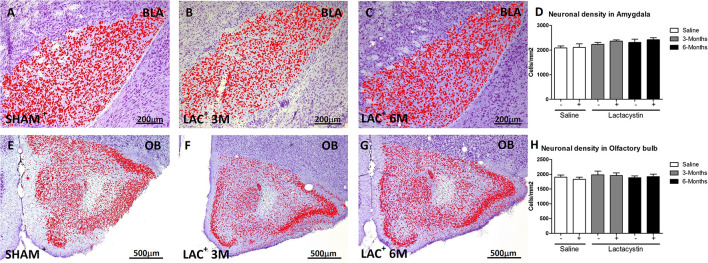
Proteasome inhibition does not induce neuronal density alterations in the BLA and OB. Representative images of Nissl-stained hemispheres injected with saline or lactacystin in the **(A–C)** BLA or **(E–G)** OB are shown. In both groups, no changes in cellular density were observed **(D,H)**. Six animals were used per group. A two-way ANOVA and Bonferroni multiple comparisons were performed to determine which pairs were significantly different (*p* < 0.05), and a two-tailed unpaired Student’s *t*-test was performed to determine differences (*p* < 0.05) between two hemispheres of the same experimental group. A confidence level of 95% was accepted as significant. No significant differences (*p* < 0.05) between two hemispheres of the same experimental group or injected hemisphere and its corresponding sham group was observed.

Brain sections were examined under light microscopy and histological analysis revealed the tips of injection needles. To assess whether LAC induces a neuronal loss in the injected areas, neuronal density in BLA and OB was analyzed. No changes in neuronal density were observed in the injected hemisphere of BLA of LAC-3M (Figures 2B,D) or LAC-6M mice (Figures 2C,D) compared with non-injected hemisphere or control mice (Figures 2A,D). Also, no changes in neuronal density were observed in the OB-injected groups (Figures 2E–H).

**FIGURE 3:**
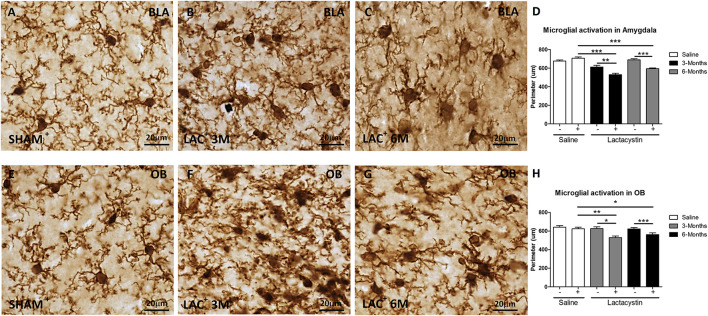
Lactacystin induces microglial activation in the BLA and OB. Representative images of microglial cells stained with IBA-1 in the injected **(A–C)** BLA or **(E–G)** OB are shown. In the BLA and OB, microglial activation at 3 (**B,F**, respectively) and 6 months (**C,G**, respectively) was observed. This change is reflected by a decrease in perimeter, as shown in **(D,H)**. Six animals were used per group. A two-way ANOVA and Bonferroni multiple comparisons were performed to determine which pairs were significantly different (*p* < 0.05), and a two-tailed unpaired Student’s *t*-test was performed to determine differences (*p* < 0.05) between two hemispheres of the same experimental group. A confidence level of 95% was accepted as significant. *Is for *p* < 0.05; **is for *p* < 0.01; ***is for *p* < 0.001.

Next, the effects of LAC on microglia and astrocyte physiology were assessed. A decrease of microglial perimeter (Figure 3D; *p* < 0.01), reflecting microglial activation, was already observed in the BLA injected hemisphere of LAC-3M mice (Figure 3B) in comparison with no injected side or control group (Figures 3A,D). At 6 months, microglia remained activated (*p* < 0.001; Figure 3D) as observed in the LAC-6M group showing one more activated and amoeboid morphology (Figure 3C) compared with more ramified surveillant microglia observed in non-injected hemispheres and control mice (Figures 3A,D).

Similar results were observed in mice injected in the OB. LAC already induced a tendential decrease of the microglial perimeter in the LAC-3M mice (Figures 3F,H) that became clear and significant at 6 months (*p* < 0.01; Figures 3G,H). Resting microglia were predominantly observed in the non-injected hemispheres and control mice (Figures 3E,H).

**FIGURE 4:**
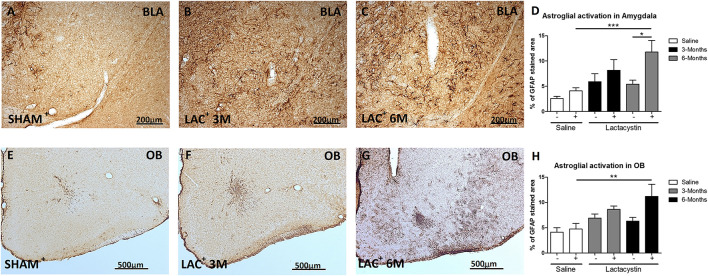
Expression of glial fibrillary acidic protein (GFAP) is increased in the BLA and OB after lactacystin injection. Representative images of astrocytes in the injected **(A–C)** BLA or **(E–G)** OB are shown. In the BLA and OB, an increased GFAP expression at (**C,G**, respectively) 6 months but not at (**B,F**, respectively) 3 months was observed. This change was quantified in **(D,H)**. Six animals were used per group. A two-way ANOVA and Bonferroni multiple comparisons were performed to determine which pairs were significantly different (*p* < 0.05), and a two-tailed unpaired Student’s *t*-test was performed to determine differences (*p* < 0.05) between two hemispheres of the same experimental group. A confidence level of 95% was accepted as significant. *Is for *p* < 0.05; **is for *p* < 0.01; ***is for *p* < 0.001.

A slight but not significant astrocyte activation (GFAP) was observed in the hemisphere of LAC-3M mice injected with LAC in comparison with the healthy hemispheres or control mice (Figure 4B). Indeed, significant astrocyte activation was only observed in the injected BLA of LAC-6M mice, showing a much more GFAP-stained area (*p* < 0.05; Figures 4C,D) than the control mice and non-injected hemispheres (Figures 4A,D).

A similar trend and results between groups were observed in the expression of GFAP in the animals injected in the OB (Figures 4E–H).

### Proteasome Inhibition Does Not Increase α-Synuclein and Phosphoser129-α- Synuclein in the Basolateral Amygdala or Olfactory Bulb

**FIGURE 5:**
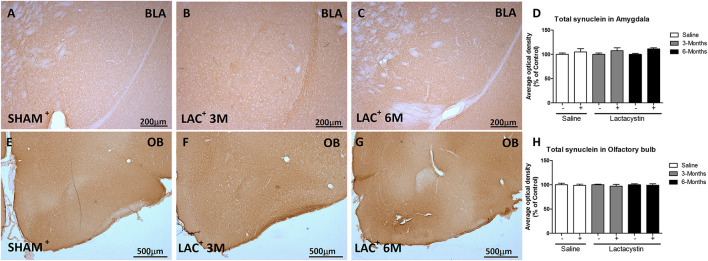
Proteasome inhibition does not increase α-synuclein (α-syn) in the BLA and OB. Representative images of hemispheres injected with saline or lactacystin in the **(A–C)** BLA or **(E–G)** OB and stained with α-syn are shown. In both groups, no changes in α-syn expression were observed **(D,H)**. Six animals were used per group. A two-way ANOVA and Bonferroni multiple comparisons were performed to determine which pairs were significantly different (*p* < 0.05), and a two-tailed unpaired Student’s *t*-test was performed to determine differences (*p* < 0.05) between two hemispheres of the same experimental group. A confidence level of 95% was accepted as significant. No significant differences (*p* < 0.05) between two hemispheres of the same experimental group or injected hemisphere and its corresponding sham group was observed.

**FIGURE 6:**
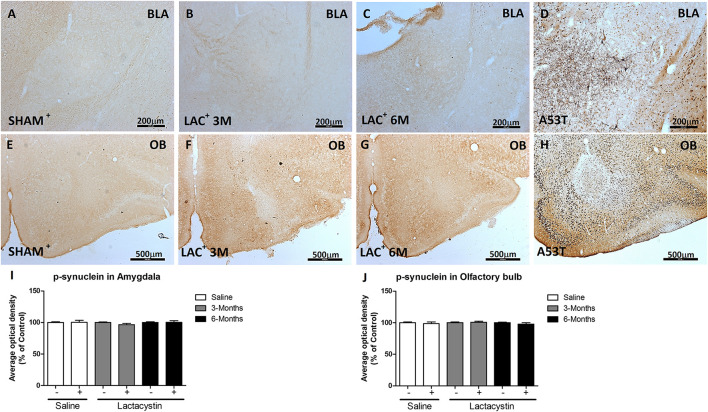
Proteasome inhibition does not increase α-syn phosphorylation in the BLA and OB. Representative images of hemispheres injected with saline or lactacystin in the **(A–C)** BLA or **(E–G)** OB and stained with phosphoSer129-α-synuclein are shown. In both groups, no changes in phosphoSer129-α-synuclein expression were observed **(I,J)**. As a positive control, images of A53T transgenic (Tg) mice overexpressing α-syn in **(D)** BLA and **(H)** OB are shown. A two-way ANOVA and Bonferroni multiple comparisons were performed to determine which pairs were significantly different (*p* < 0.05), and a two-tailed unpaired Student’s *t*-test was performed to determine differences (*p* < 0.05) between two hemispheres of the same experimental group. A confidence level of 95% was accepted as significant. No significant differences (*p* < 0.05) between two hemispheres of the same experimental group or injected hemisphere and its corresponding sham group was observed.

To study whether the inhibition of proteasome with LAC leads to the accumulation of α-syn or pS129-α-syn, immunohistochemistry against both proteins in the BLA (Figures 5A–D, 6A–D) and OB (Figures 5E–H, 6E–H) was performed and quantified as detailed in methods. As a positive control, sections of A53T transgenic (Tg) mice overexpressing α-synuclein were included (Figures 6D,H). In both regions, no changes in the expression of α-syn or pS129-α-syn between the LAC and control groups were observed (Figures 5D,H, 6I,J), whereas the levels of pS129-α-syn staining in such groups were significantly lower when compared with sections of the BLA and OB of A53T-Tg mice overexpressing pS129-α-syn (Figures 6I,H, respectively).

### Expression of Nigrostriatal Dopamine Remains Unaltered After Lactacystin Injection in the Basolateral Amygdala or Olfactory Bulb

**FIGURE 7:**
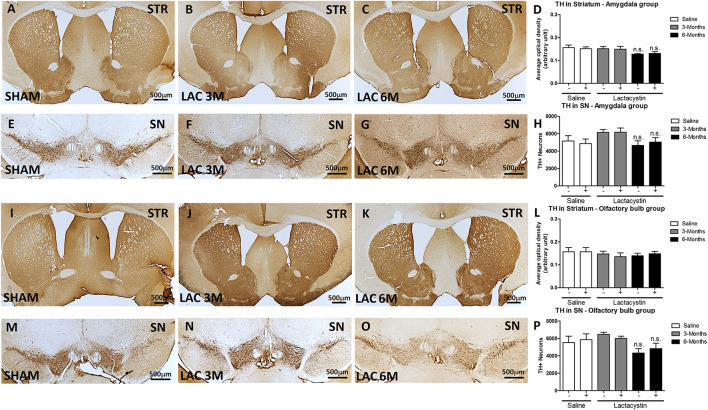
Expression of nigrostriatal dopamine (DA) remains unaltered after lactacystin injection in the BLA or OB. Representative images and graphs showing no changes in striatal dopaminergic terminals in mice injected into the **(A–D)** BLA or **(I–L)** OB are shown. No differences were also observed in dopaminergic neurons in the SNc in mice injected in the **(E–H)** BLA or **(M–P)** OB. Six animals were used per group. A two-way ANOVA and Bonferroni multiple comparisons were performed to determine which pairs were significantly different (*p* < 0.05), and a two-tailed unpaired Student’s *t*-test was performed to determine differences (*p* < 0.05) between two hemispheres of the same experimental group. A confidence level of 95% was accepted as significant. No significant differences (*p* < 0.05) between two hemispheres of the same experimental group or injected hemisphere and its corresponding sham group was observed.

Finally, the effects of proteasome inhibition on neurons in the SNc and its dopaminergic terminals in the striatum were assessed. No changes in striatal dopaminergic terminals were observed in either of the injected groups. Optical density analysis revealed similar values between the LAC and control groups of mice injected in the BLA (Figures 7A–D) or OB (Figures 7I–L).

Similarly, proteasome inhibition with LAC does not produce alterations in DA neurons in the SN. In fact, the stereological analysis showed no differences in the number of TH + immunopositive cells between the LAC and control groups of mice injected in the BLA (Figures 7E–H) or OB (Figures 7M–P).

## Discussion

Proteasomal dysfunction has been long proposed to be involved as a potential contributor to neurodegeneration in PD (McNaught and Jenner, 2001; McNaught et al., 2002b, 2003; Tofaris et al., 2003; Grünblatt et al., 2004; Lim and Tan, 2007; Bukhatwa et al., 2010; Ebrahimi-Fakhari et al., 2012). Dysfunctional proteasomes can result in aberrant protein turnover and accumulation of misfolded proteins, such as α-syn (McNaught et al., 2001; Olanow and McNaught, 2006; Ebrahimi-Fakhari et al., 2012). Testing for proteasome inhibition typically requires the use of highly specific and potent inhibitors. One of the most used is LAC (Bentea et al., 2017; Ômura and Crump, 2019). LAC has been proven to lead to α-syn pathology *in vitro* and *in vivo* (McNaught et al., 2002a; Miwa et al., 2005; Vernon et al., 2010; Mackey et al., 2013; Pienaar et al., 2015b; McKinnon et al., 2020). Correspondingly, proteasomal inhibitors given in combination with other classic PD neurotoxins, such as MPP+, rotenone, or 6-OHDA, enhance the formation of α-syn-positive inclusions in dopaminergic neurons (Sawada et al., 2004; Inden et al., 2005).

Previous evidence has shown that LAC could lead to retrograde accumulation of α-syn *in vivo* (Laser et al., 2003; Miwa et al., 2005) and ubiquitin- and α-syn-positive inclusions in both TH+ and non-TH+ neurons *in vitro* and *in vivo*, suggesting that the accumulation of α-syn can occur in non-dopaminergic neurons after injection of LAC or other proteasome inhibitors (Rideout et al., 2005; Ebrahimi-Fakhari et al., 2011; Bentea et al., 2015). In the same way, intranasal administration of high levels of LAC led to a decrease in the number of dopaminergic neurons in the OB and SNc of rats (Ekimova et al., 2016; Ekimova and Plaksina, 2017). Thus, we hypothesized whether long-term proteasome inhibition in regions of the brain of patients with PD that are more disposed to α-syn pathologies, such as the OB and amygdala, could lead to long-term α-syn accumulation and dopaminergic degeneration, as has been described with other toxic species (monomers, oligomers, fibrils, AAV-based, etc.) (Flores-Cuadrado et al., 2019; Heras-Garvin and Stefanova, 2020; Fares et al., 2021).

In this proof-of-concept study, injection in the OB and BLA did not produce any parkinsonian-related pathological effects on the WT mice up to 6 months post-injection. We did not observe changes in neuronal density, ubiquitin, or significant accumulation of α-syn at the sites of injection up to 6 months. In several studies, increased α-syn has been observed 1 week after LAC injection in the SNc (Bentea et al., 2015; Savolainen et al., 2017), whereas in this study we waited up to 3 and 6 months. While we were expecting a significant gradual increase in the effect of α-syn accumulation over time, it may also be that those acute pathological effects occurring early (weeks) disappeared or were resolved or compensated after a long period of time. The degradation of numerous proteins, such as α-syn, linked to neurodegenerative diseases was not significantly retarded by proteasomal inhibitors, suggesting that after an initial delay, the degradation of some proteins could take alternative routes (Hakim et al., 2016). For example, proteasome inhibition can induce a compensatory activation of the autophagy-lysosomal pathway, which would diminish the long-term effects of LAC injections (Ding and Keller, 2003; Rideout et al., 2004; Shen et al., 2013). Consistent with this, recent studies with intracerebroventricular injections in minipigs showed transient dopaminergic effects observed by PET that was not sustained at 6 months. In addition, no differences in α-syn accumulation or dopaminergic markers at the striatum, SNc, or locus coeruleus up to 6 months were observed, but there was mild inflammation, all in agreement with our results (Lillethorup et al., 2018a). Similarly, systemic administration of PSI only transiently decreased brain proteasome activity in the absence of any evidence of lesions of nigrostriatal dopamine neurons or locomotor activity (Mathur et al., 2007). It is also possible that neurons in the SNc are more vulnerable to LAC-induced α-syn accumulation. Preferential toxicity of proteasomal inhibitors toward dopaminergic cells has been demonstrated before (Mytilineou et al., 2004; Reaney et al., 2006). Supporting this concept, structural and functional defects in the 26/20S proteasome seems to be specific for the SNc, and do not occur in other regions such as the striatum or various cortical regions, where there is no neuronal loss in patients with PD (Furukawa et al., 2002; Tofaris et al., 2003; Grünblatt et al., 2004). Other studies did not find significant α-syn accumulation after systemic LAC administration or local injections in regions other than the SNc (Lorenc-Koci et al., 2011; Bentea et al., 2017). For example, Lorenc-Koci compared intranigral and intrastriatal injection of lactacystin in rats, showing absence of alterations in the latter (Lorenc-Koci et al., 2011).

Importantly, we observed increased inflammation measured as astroglial and microglial cell activation in both the OB and BLA, which is in agreement with previous reports showing neuroinflammation after local LAC injections (Bentea et al., 2015; Elson et al., 2016; Savolainen et al., 2017; Lillethorup et al., 2018b; Deneyer et al., 2019). Interestingly, we observed increasingly significant neuroinflammation at 3 and 6 months, probably indicating long-term effects of local proteasome inhibition or a combined effect of defective proteasome system with aging. We eventually analyzed the integrity of the nigrostriatal pathway, showing that there was no loss of dopaminergic neurons in the SNc or striatal fibers in the striatum, which is in agreement with the behavioral analysis, where no motor signs were obvious up to 6 months. Intriguingly, decreased number of dopaminergic neurons in the OB and SNc without any motor behavior disorders were observed in one study after intranasal administration of LAC in rats (Ekimova et al., 2016). However, the doses used in that study were much higher, 500 μg, and administered over a longer period, while we only used 4 μg for a single injection. Noteworthy, the paradigm used in this study regarding lactacystin concentration and volume of injection are in the range of those that produce pathology in the SNc in other studies (Lorenc-Koci et al., 2011; Bentea et al., 2015). It might be that different ways of administration, the region injected, and differences between species are crucial to cause neurodegeneration.

Overall, we could not find significant parkinsonian-related changes after LAC injections in the OB and BLA after 6 months. Before, proteasome inhibition as the model for PD has led to conflicting results especially when the injection was administered systemically (Bentea et al., 2017). While some studies showed an increase in α-syn, others found that α-syn protein levels in the SNc were not affected even by high doses of LAC injected intrastriatally (Lorenc-Koci et al., 2011). Even more, some studies showed that proteasome inhibitors protect against other toxins such as MPTP or 6-OHDA (Inden et al., 2005; Oshikawa et al., 2009). Similarly, while some studies suggest that inhibition of proteasome activity in the amygdala impairs long-term memory (Lopez-Salon et al., 2001; Artinian et al., 2008; Jarome et al., 2011), others have found that proteasome inhibitors have no effect (Lee et al., 2008) or even enhance memory (Yeh et al., 2006; Felsenberg et al., 2012).

This study has some limitations: First, sex can be a biological variable. In the current experimental design, we used male mice because most of the studies using similar approaches involved male mice. Sexual dimorphism in proteasomal inhibition remains an area for future investigations. Second, for the same reason, we decided to perform unilateral injections in order to use the contralateral hemisphere as control. It could be that a bilateral paradigm would induce significant behavioral differences, although this was out of the scope of this preliminary study, and the motor tests were consistent with the fact that the mice did not have dopaminergic deficits. The fact that there is an increase of inflammation markers only in the structure of the hemispheres injected with LAC and not with saline probably indicates that LAC was well injected, as inflammation has been widely demonstrated after LAC injections (Bentea et al., 2015; Elson et al., 2016; Savolainen et al., 2017; Lillethorup et al., 2018b; Deneyer et al., 2019). Finally, in this exploratory study, we have chosen the OB and amygdala as extra-striatal regions, because both of them exhibit high α-syn pathology consistently in all patients with PD, and there is plenty of literature involving these regions in other α-syn models. However, it is possible that injections in other structures, as what happens in the SNc, might behave differently; therefore, our results might not be generalized to all peripheral structures.

Here, we add to the increasing body of literature showing that LAC injections in extra-nigrostriatal regions may not be useful in modeling certain specific aspects of PD such as α-syn spreading after a long period of time. By showing the different vulnerability of distinctive regions, this study also reinforces the especial and extraordinary vulnerability of the dopaminergic neurons of the SNc to diverse insults.

## Data Availability Statement

The raw data supporting the conclusions of this article will be made available by the authors, without undue reservation.

## Ethics Statement

The animal study was reviewed and approved by HM CINAC Ethical Committee on Animal Welfare and Care.

## Author Contributions

JB and IT-D designed the study. NL-GR, TB, LM-R, and CS-R performed and analyzed the experiments. NL-GR, TB, IT-D, AR-F, LA-E, and JB interpreted the data and wrote the manuscript. All authors critically revised the manuscript, read and agreed to the published version of the manuscript.

## Conflict of Interest

The authors declare that the research was conducted in the absence of any commercial or financial relationships that could be construed as a potential conflict of interest.

## Publisher’s Note

All claims expressed in this article are solely those of the authors and do not necessarily represent those of their affiliated organizations, or those of the publisher, the editors and the reviewers. Any product that may be evaluated in this article, or claim that may be made by its manufacturer, is not guaranteed or endorsed by the publisher.

## Supplementary Material

Supplementary Figure 1 The expression of ubiquitin remains unaltered in basolateral amygdala and olfactory bulb after 3 and 6 months of lactacystin injection. Representative images of ubiquitin in the injected BLA **(A–C)** or OB **(E–G)** are shown. In both groups, no changes in ubiquitin expression were observed (**D** and **H**). 6 animals per group were used. A two-way ANOVA and Bonferroni multiple comparisons were used to determine which pairs were significantly different (*p* < 0.05) and a two-tailed unpaired Student’s *t*-test was used to determine differences (*p* < 0.05) between two hemispheres of the same experimental group. A confidence level of 95% was accepted as significant. No significant differences (*p* < 0.05) between two hemispheres of the same experimental group or injected hemisphere and its correspondent sham groups were observed.
