# Supplementary figures and images for: Lack of Parkinsonian Pathology and Neurodegeneration in Mice After Long-Term Injections of a Proteasome Inhibitor in Olfactory Bulb and Amygdala
Source: Front Aging Neurosci. 2021 Oct 21;13:698979. doi: 10.3389/fnagi.2021.698979 (PMC8570189; doi:10.3389/fnagi.2021.698979)

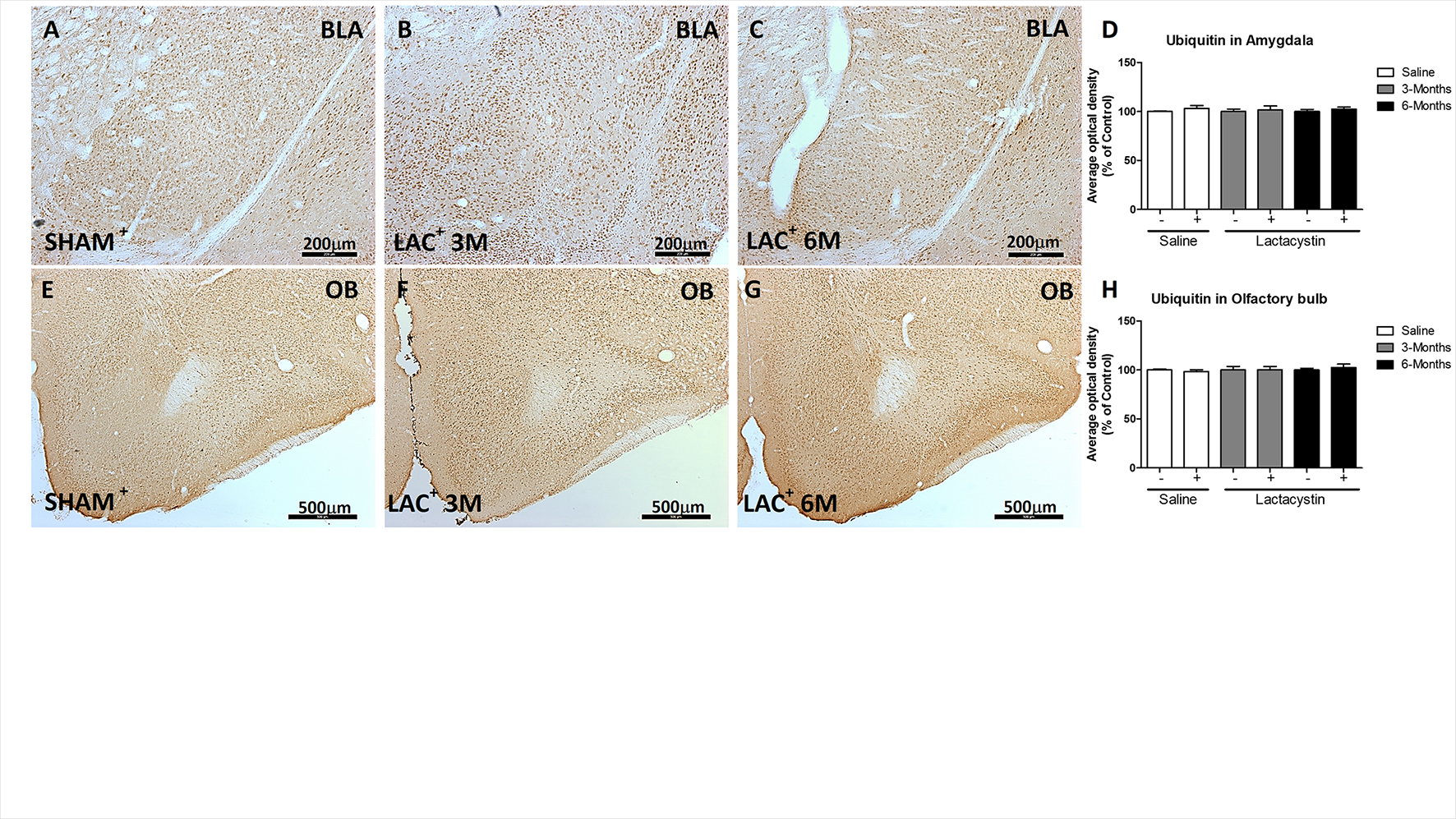

Supplement: Supplementary Figure 1 — The expression of ubiquitin remains unaltered in basolateral amygdala and olfactory bulb after 3 and 6 months of lactacystin injection. Representative images of ubiquitin in the injected BLA (A–C) or OB (E–G) are shown. In both groups, no changes in ubiquitin expression were observed (D and H). 6 animals per group were used. A two-way ANOVA and Bonferroni multiple comparisons were used to determine which pairs were significantly different (p < 0.05) and a two-tailed unpaired Student’s t-test was used to determine differences (p < 0.05) between two hemispheres of the same experimental group. A confidence level of 95% was accepted as significant. No significant differences (p < 0.05) between two hemispheres of the same experimental group or injected hemisphere and its correspondent sham groups were observed. [file Image_1.TIF]
